# Venom Peptides Across Asian and American Tarantulas Utilize Dual Pharmacology to Target Activation and Fast Inactivation of Voltage-Gated Sodium Channels

**DOI:** 10.3390/toxins17110561

**Published:** 2025-11-14

**Authors:** Amatulla S. Nashikwala, Charan Kotapati, David A. Eagles, Richard J. Lewis, Fernanda C. Cardoso

**Affiliations:** 1 Institute for Molecular Bioscience, The University of Queensland, St Lucia, Brisbane, QLD 4072, Australia; 2 Centre for Motor Neuron Disease Research, Faculty of Health, Medicine and Behavioural Sciences, The University of Queensland, St Lucia, Brisbane, QLD 4072, Australia; 3 Centre for Innovation in Pain and Health Research, Faculty of Health, Medicine and Behavioural Sciences, The University of Queensland, St Lucia, Brisbane, QLD 4072, Australia

**Keywords:** spider peptide, sodium channels, structure-function relationship, dual pharmacology, neurological disorders, novel therapies

## Abstract

Spider-derived venoms are a rich source of cystine knot peptides with immense therapeutic potential. Many of these peptides exert unique biological activities through the modulation of ion channels, including of human voltage-gated sodium (Na_V_1.1–Na_V_1.9) channels. Na_V_ channel subtypes have diverse functions determined by their tissue and cellular distribution and biophysical properties, and are pathophysiology mediators in various diseases. Therefore, Na_V_s are central in studies of human biology. This work investigated the pharmacological properties of venom of the Thai theraphosid *Ornithoctonus aureotibialis* on Na_V_ channels. We discovered a predominant venom peptide named Oa1a and assessed its pharmacological properties across human Na_V_ channel subtypes. Synthetic forms of the peptide Oa1a showed preferential inhibition of Na_V_1.1 and Na_V_1.7, while recombinant Oa1a displayed a preference for inhibiting Na_V_1.2, Na_V_1.6, and Na_V_1.7. Interestingly, all versions of Oa1a peptides exerted dual pharmacological effect by reducing the peak current and slowing fast inactivation of Na_V_1.3, consistent with Oa1a having more than one binding site on Na_V_ channels. Such complex pharmacology was previously observed for a venom peptide in a Central American and Costa Rican tarantula, suggesting a conserved mechanism of action amongst these geographically distinct species. However, Oa1a lacked activity in the T-type channels observed in the tarantula peptide from Central America. Structure–function relationships investigated using molecular modelling showed that the dual pharmacology is driven by a conserved mechanism utilizing a mix of aromatic and charged residues, while the T-type activity appears to require additional charged residues in loop 2 and fewer positive charges in loop 4. Future structure–activity relationship studies of Oa1a will guide the development of pharmacological tools as well as next-generation drugs to treat Na_V_ channel dysfunction associated with neurological disorders.

## 1. Introduction

Venoms comprise a rich source of biologically active peptides that enable the capture of prey and deterrence of predators by venomous animals [1,2]. Amongst those, spiders, scorpions, snakes, cone snails, and sea anemones produce venoms containing small molecules, peptides, and proteins that possess pharmacological properties on an array of membrane receptors and ion channels, and with the potential to become novel drugs and bioinsecticides [3,4,5,6]. Spider venoms, in particular, are an extraordinary source of cystine-knot peptides (so-called inhibitory cysteine knot (ICK) peptides or ‘knottins’), many of which are potent and selective modulators of voltage-gated sodium (Na_V_) channels [7,8,9].

Na_V_ channels play a pivotal role in the initiation and propagation of electrical signals in excitable cells and, consequently, are involved in the etiology of many neurological disorders, including pain, epilepsy, and neurodegeneration [10,11]. The subtypes Na_V_1.1, Na_V_1.3, and Na_V_1.6–1.9 have been implicated in the pathophysiology of neuropathic pain [12], as have the subtypes Na_V_1.1–1.3 and Na_V_1.6 in epilepsy [11]. In addition, the subtypes Na_V_1.2–1.3 and Na_V_1.6 have been implicated in aberrant hyperexcitability leading to neurodegeneration in diseases such as amyotrophic lateral sclerosis [13,14,15].

Venom peptides are unique pharmacological tools utilized in studies of Na_V_ dysfunction in disease as well as lead candidates for drug development. This was demonstrated by the venom peptide Tap1a applied in studies of chronic visceral pain [16] and Hm1a in studies of epilepsy [17]. Such remarkable pharmacological properties highlight venoms as a treasure trove of bioactive compounds with the potential to create novel pharmacological tools assisting in investigations of pathophysiological mechanisms in complex neurological diseases and with rich applications in drug candidates.

Spider venom components target specific sites on the Na_V_ channel, often site 2 located in domain IV and/or site 4 located in domain II of the channel [18]. The site of action defines the venom peptide’s pharmacological properties, which can act as an inhibitor by targeting site 4 [19] or as an activator by targeting site 2 [20]. In some cases, spider peptides have high affinity for both sites and produce a mixed pharmacology modulating activation and fast inactivation, which is characteristic of Na_V_ subtype-selective peptides [21].

In this work, we investigated the pharmacological potential of the venom from the Asian Thai tarantula *Ornithoctonus aureotibialis* in modulating Na_V_ channels and reported the identification of a new spider venom peptide named Oa1a with pharmacological properties acting as a dual modulator of Na_V_ channels. We produced Oa1a using recombinant expression and chemical synthesis and performed a full characterization of its pharmacological properties using electrophysiological measurement on the Na_V_1.1 to Na_V_1.7 channels. Synthetic Oa1a showed a strong preference for inhibiting Na_V_1.1 and Na_V_1.7, while recombinant Oa1a displayed a preference for inhibiting Na_V_1.2, Na_V_1.6, and Na_V_1.7. In addition, Oa1a showed strong dual modulation of Na_V_1.3 followed by Na_V_1.1 and Na_V_1.5. Such pharmacological properties resemble dual modulators previously described in a tarantula endemic of Central America, demonstrating conserved venom pharmacology in geographically far-apart tarantula species.

## 2. Results

### 2.1. Discovery of Oa1a Peptide

**Figure 1:**
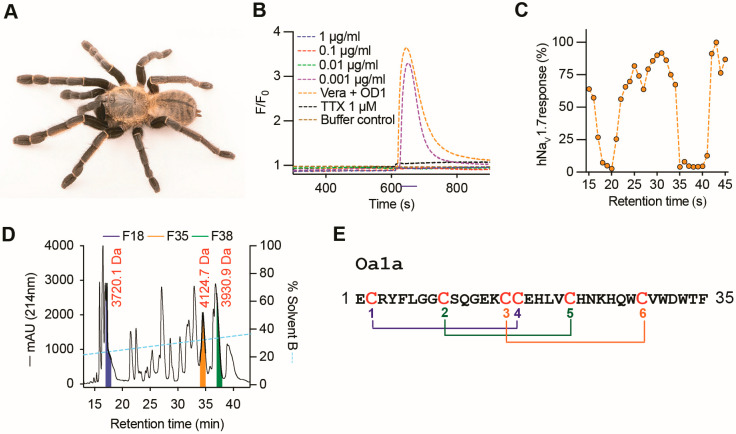
The venom of *Ornithoctonus aureotibialis* modulates Na_V_ channels. (**A**) Photo of the tarantula *Ornithoctonus aureotibialis* (01707252 ©Joel Sartore/Photo Ark/naturepl.com). (**B**) Fluorescence-imaging assay using neuroblastoma SH-SY5Y cells and [Ca^2+^]_i_ measurements showed crude venom inhibited Na_V_-mediated [Ca^2+^]_i_ responses at 1 to 0.01 μg/mL, and lost inhibitory activity at 0.001 μg/mL. (**C**) Venom fractions evaluated using the same fluorescence-imaging assay revealed venom components engendering Na_V_ inhibition were eluted in the retention times 18–20 min and 35–41 min. (**D**) Venom fractionation RP-HPLC chromatogram and mass spectrometry analysis showed peptides with molecular weight 3720.1 Da in fraction 18 (F18), 4124.7 Da in fraction 35 (F35), and 3930.9 Da in fraction 38 (F38). (**E**) N-terminal Edman degradation analysis of F35 revealed the sequence of Oa1a with 35 residues comprising 6 cysteines arranged in a typical scaffold of an inhibitory cysteine knot peptide from spider venom peptides.

The venom from the tarantula *Ornithoctonus aureotibialis* (Figure 1A) produced a strong inhibitory response on human Na_V_1.7 at 1, 0.1, and 0.01 μg/mL concentrations, assayed using a fluorescence-imaging plate reader assay and intracellular calcium measurements, with loss of inhibitory activity at 0.001 μg/mL concentration (Figure 1B). Venom components separated using RP-HPLC and eluted between 18 and 20 min and 35 to 40 min retention time showed strong inhibitory activity on human Na_V_1.7 using this same fluorescent calcium imaging assay (Figure 1C). Mass spectrometry analysis revealed three major peptide masses likely involved in the inhibitory activity observed, with masses 3720.1 (fraction 18), 4124.7 (fraction 35), and 3930.9 Da (fraction 37) (Figure 1D). In this work, the peptide in fraction 35 with a mass of 4124.7 Da was submitted to N-terminal sequencing using Edman degradation, revealing a primary structure with 34 residues and the expected predicted mass of 4124.7 Da (Figure 1E). This new spider venom peptide was named Oa1a and displayed six cysteines arranged in the typical inhibitory cysteine knot scaffold found in spider venom peptides [7].

### 2.2. Production of Recombinant and Synthetic Oa1a

**Figure 2:**
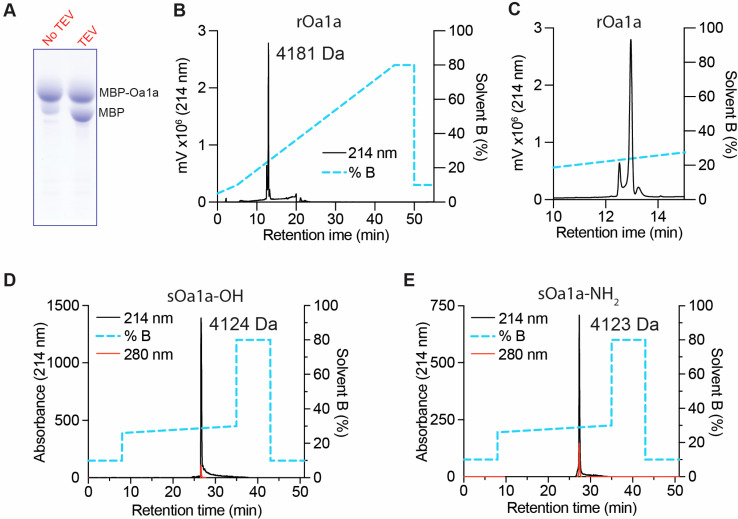
Production of the spider peptide Oa1a. (**A**) SDS-Page of the production of recombinant Oa1a (rOa1a) fused to the maltose binding protein (MBP) using a bacterial expression system and released from the MBP fusion using Tobacco Etch Virus (TEV) protease cleavage. The fusion of MBP-Oa1a has a higher molecular weight than MBP alone. (**B**) Chromatogram of the rOa1a purified using RP-HPLC in C18 column. The peptide had the expected molecular weight 4181 Da which has an additional N-terminal glycine reminiscent of the TEV cleavage site. (**C**) rOa1a eluted at room temperature presented two isoforms characteristic of other spider peptides such as Df1a [21]. (**D**,**E**) Chromatogram of the synthetic Oa1a-OH (**D**) and Oa1a-NH2 (**E**) purified using RP-HPLC in C18 column. Oa1a-OH presented the expected molecular weight 4124 Da, while Oa1a-NH_2_ presented the expected molecular weight 4123 Da. Both synthetic peptides were eluted as single isomer at 40 °C.

The peptide Oa1a was produced via recombinant expression as an MBP-Oa1a fusion protein and released from the fusion using TEV protease cleavage in an oxidative buffer containing reduced and oxidized glutathione (Figure 2A). Recombinant Oa1a (rOa1a) was purified using RP-HPLC in C18 column and acetonitrile gradient, yielding a recombinant peptide displaying the expected molecular weight 4181 Da for the native peptide with an additional N-terminal glycine reminiscent of the TEV protease cleavage site (Figure 2B). The recombinant Oa1a was eluted as two isomers at room temperature (Figure 2C) as previously observed for other similar spider peptides such as Df1a [21].

Synthetic Oa1a (sOa1a) was produced in the C-terminal carboxyl or amidated forms using solid-phase synthesis, oxidized in reduced and oxidized glutathione to form the expected six cysteine bridges, and purified using RP-HPLC in C18 column and acetonitrile gradient (Figure 2D,E). Both sOa1a-OH and sOa1a-NH_2_ were eluted as a single isomer upon purification at 40 °C, similarly to the previously described peptide Df1a [21]. The expected molecular masses were observed for the sOa1a with C-terminal carboxyl with mass 4124 Da, and the sOa1a with the C-terminal amide with mass 4123 Da (Figure 2D,E).

### 2.3. The Venom Peptide Oa1a Modulates Na_V_ Channels

**Figure 3:**
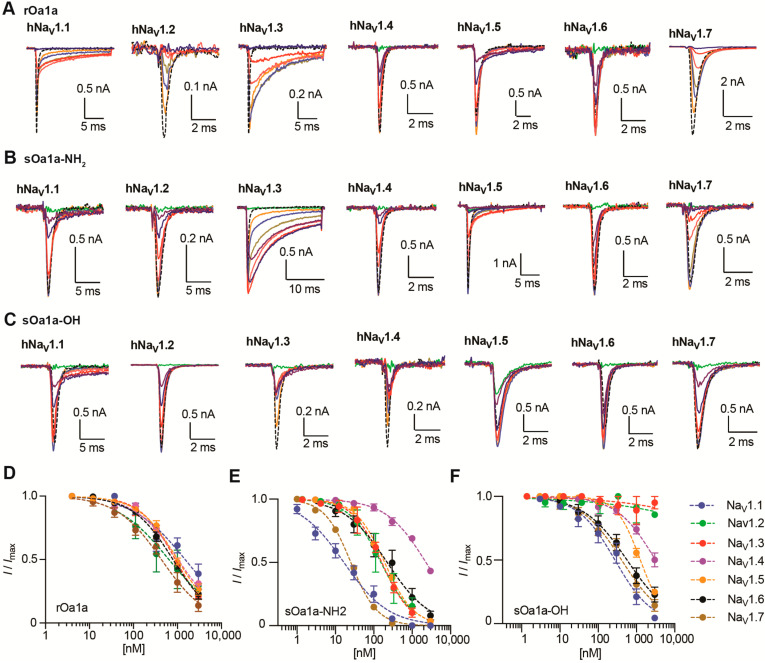
Na_V_ channel subtype pharmacological profiles of rOa1a, sOa1a-NH_2_, and sOa1a-OH determined using automated electrophysiology. (**A**–**C**) Representative current traces from the hNa_V_1.1 to hNa_V_1.7 channels in the presence of increasing concentrations of rOa1a (**A**), sOa1a-NH_2_ (**B**) and sOa1a-OH (**C**) peptides measured by automated whole-cell patch clamp in QPatch 16X. Holding potential was −80 mV and Na^+^ currents were elicited by a 20 ms voltage step to 0 mV from a −120 mV conditioning pulse applied for 200 ms. A strong slowing in the fast inactivation of Na_V_1.3 was observed and was further analyzed later in this work. (**D**–**F**) Dose responses for the inhibition of the hNa_V_1.1 to hNa_V_1.7 channels in the presence of increasing concentrations of rOa1a (**D**), sOa1a-NH_2_ (**E**) and sOa1a-OH (**F**) peptides. The IC_50_ values calculated from these dose responses are described in Table 1.

The recombinant and synthetic forms of Oa1a were evaluated for their pharmacological properties on Na_V_1.1 to Na_V_1.7 channel subtypes using automated whole-cell patch clamp electrophysiology and a closed-state voltage protocol as previously described by us [21,22]. The rOa1a showed a preference for inhibiting Na_V_1.2, Na_V_1.3, Na_V_1.6, and Na_V_1.7 at a higher nanomolar range, with IC_50_ values (in nM) of 640, 899, 792, and 432, respectively, followed by IC_50_ values (in nM) for Na_V_1.1, Na_V_1.4, and Na_V_1.5 above 1 μM (Table 1, Figure 3A,D). Whole-cell patch clamp current traces showed that rOa1a inhibited the peak current as well as strongly slowing the fast inactivation of Na_V_1.3 (Figure 3A).

Both synthetic forms of Oa1a-NH_2_ and Oa1a-OH showed strong preference for inhibiting Na_V_1.1, with IC_50_ values (in nM) of 17 and 274, respectively, followed by Na_V_1.7 with IC_50_ values (in nM) of 25 and 410, and Na_V_1.6 with IC_50_ values (in nM) of 223 and 568, respectively (Table 1, Figure 3B,C,E,F). Synthetic Oa1a-NH_2_ had strong inhibitory activity on Na_V_1.2, Na_V_1.3, and Na_V_1.5 with IC_50_ values (in nM) of 154, 162, and 192, and weak activity on Na_V_1.4 with an IC_50_ value of 2.3 μM (Table 1, Figure 3B,E). On the other hand, Oa1a-OH had weak activity on Na_V_1.5 with an IC_50_ value of 1.26 μM and no activity on Na_V_1.2-Na_V_1.4 when tested at up to 3 μM (Table 1, Figure 3C,F). Like rOa1a, sOa1a-NH_2_ also displayed dual modulatory activity by strongly slowing the fast inactivation of Na_V_1.3 (Figure 3B). Interestingly, this dual modulatory activity was not observed in sOa1a-OH at the concentrations tested in this work.

**Figure 4:**
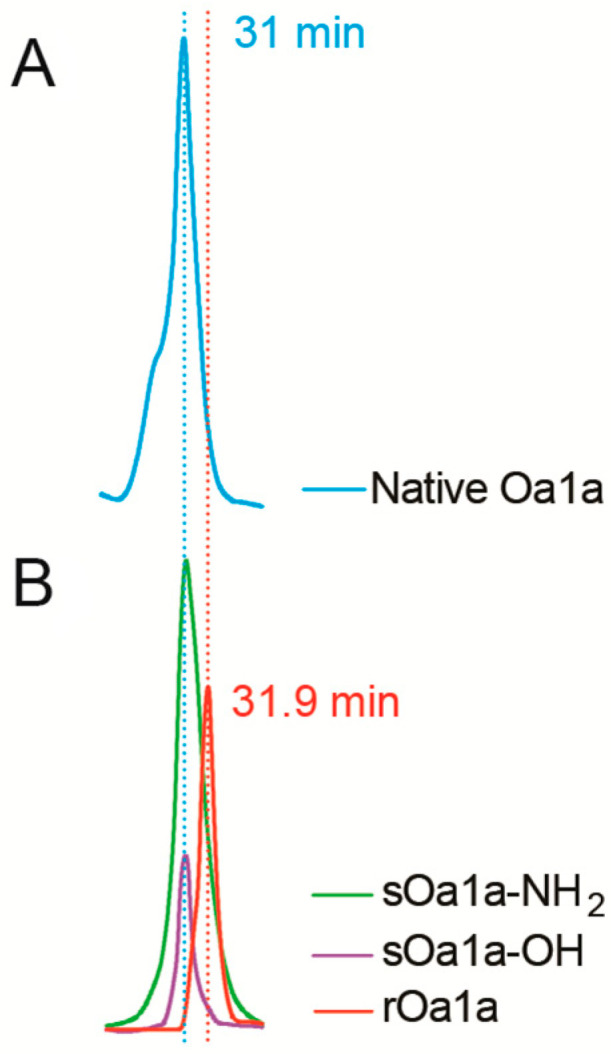
Co-elution of the native peptide Oa1a along with its recombinant and synthetic forms. (**A**) Chromatogram of the purified native Oa1a (approximately 500 ng, blue trace) using RP-HPLC in C18 column and a gradient of 10% to 50% B over a period of 30 min at 0.5 mL/mL. The native Oa1a eluted at 31 min. (**B**) Overlayed chromatograms of the purified recombinant Oa1a (1 μg, red trace) and purified synthetic Oa1a-NH_2_ (2 μg, green trace) and Oa1a-OH (500 ng, purple trace) peptides using RP-HPLC in C18 column and a gradient 10% to 50% B over a period of 30 min at 0.5 mL/mL. The synthetic versions of Oa1a eluted at 31 min, suggestive of identical three-dimensional structure compared to native Oa1a. Recombinant Oa1a had a 0.9 min delay in elution, indicative of a distinctive fold structure in relation to native and synthetic Oa1a. Chromatogram traces in (**A**,**B**) were recorded at 280 nm.

We also tested both synthetic forms of Oa1a-NH_2_ and Oa1a-OH on the T-type channel subtype Ca_V_3.2 using automated whole-cell patch clamp electrophysiology and a closed-state voltage protocol. We did not observe activity on the Ca_V_3.2 channel at up to 10 μM tested (Figure S1). The differences in potency and selectivity of the recombinant Oa1a compared to the synthetic forms of Oa1a led to further investigations into their chemical properties using co-elution experiments in RP-HPLC (Figure 4). We found that sOa1a-NH_2_ and sOa1a-OH had identical elution times compared to native Oa1a at 31 min (Figure 4A,B). On the other hand, rOa1a had a delayed elution time of 31.9 min, suggestive of a distinct structure compared to native and synthetic Oa1a (Figure 4B). This difference is reflected in their pharmacological properties and likely defined the differences in potency observed in our electrophysiology experiments.

### 2.4. Mode of Action of Oa Peptides on Na_V_ Channels

**Figure 5:**
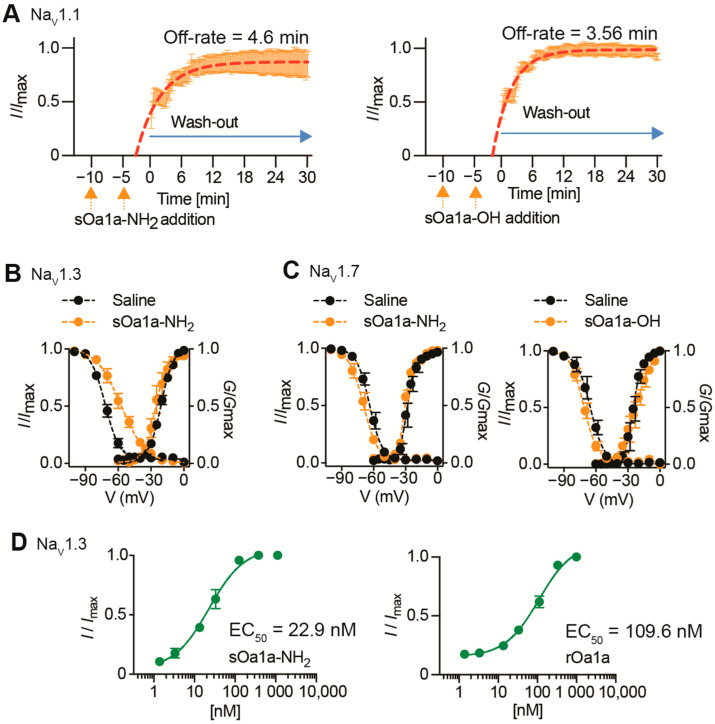
Mode of action of Oa1a at select Na_V_ subtypes. (**A**) Off-rate measurements of sOa1a-OH and sOa1a-NH_2_ over Na_V_1.1 were calculated from the fitted exponentials of experiments recorded before and after addition of peptides at 10x IC_50_ concentration followed by washes with extracellular solution. C-terminal amidation contributed to enhance irreversibility by increasing the off-rate by approximately 1 min. (**B**,**C**) Current–voltage relationship evaluations on Na_V_1.3 showed sOa1a-NH_2_ administered at IC_50_ concentration shifted the steady-state inactivation to more depolarized potentials by 14 mV, while the current–voltage relationships of Na_V_1.7 remained nearly unaltered in the presence of sOa1a-NH_2_ or sOa1a-OH administered at IC_50_ concentrations with V_50_ values lesser or equal to 5 mV. (**D**) The potency of sOa1a-NH_2_ and rOa1a in slowing the fast inactivation of Na_V_1.3 was determined, showing a potent effect at low nanomolar range for both peptides, with rOa1a showing 4.8-fold less potency compared to sOa1a-NH_2_.

We performed off-rate evaluations to investigate the reversibility properties of synthetic Oa1a-OH and Oa1a-NH_2_ (Figure 5A, Table 2). Both versions of Oa1a tested at a concentration of 10× the IC_50_ value for Na_V_1.1 were completely reversed by washing. The Oa1a-NH_2_ washed out with an off-rate of 4.6 min while Oa1a-OH washed out with an off-rate of 3.56 min, suggesting the C-terminal amidation contributed to enhancing irreversibility by increasing the off-rate by 1 min.

The evaluation of the current–voltage relationships showed that sOa1a-NH_2_ tested at IC_50_ value shifted its steady-state inactivation to more depolarized potentials with a ΔV_50_ of 14 mV (Figure 5B, Table 2), with V_50_ values (in mV, mean ± SEM) of −71 ± 0.71 for EC solution control and -57 ± 1.4 in the presence of an EC_50_ value of 23 nM sOa1a-NH_2_ (Figure 5D). This result corroborates with the strong slowing in fast inactivation observed in the closed-state electrophysiology experiments (Figure 3B). No shifts above 5 mV were observed in the voltage of activation of Na_V_1.3 in the presence of 23 nM sOa1a-NH_2_.

The evaluation of the voltage–current relationships showed sOa1a-NH_2_ tested at IC_50_ value did not alter the voltage of activation or the steady-state inactivation of the subtype Na_V_1.7 (Figure 5C, Table 2). This suggests the Na_V_ inhibitory mechanism of Oa1a is similar to other spider peptides targeting site 4 in the voltage-sensor of the DII and trapping segment 4 in the closed state position [23].

We further evaluated the potency of Oa1a in slowing the fast inactivation of Na_V_1.3 (Figure 5D, Table 2). The calculation of the EC_50_ values of sOa1a-NH_2_ and rOa1a for slowing the fast inactivation of Na_V_1.3 produced values (in nM) of 22.9 ± 1.8 and 109.6 ± 7.2, respectively (Figure 5D, Table 2). The ability of Oa1a to slow the fast inactivation of Na_V_ subtypes suggests the binding and modulation of site 2 in the voltage-sensor of the DIV of these subtypes, as previously observed for other spider peptides [21].

### 2.5. Structure–Function Relationships of Potency and Selectivity of Oa for Na_V_s

**Figure 6:**
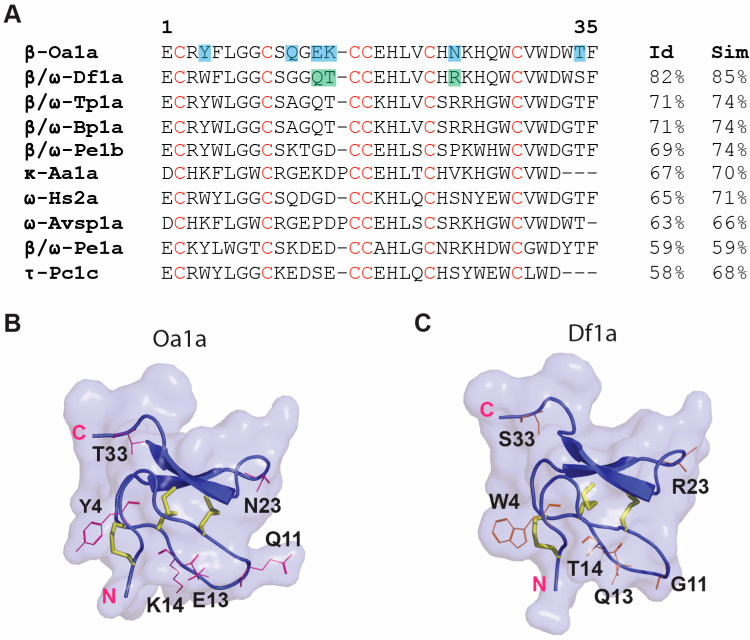
Structural analysis of β-Oa1a. (**A**) Primary sequence identity and similarity of Oa1a compared to other similar spider venom peptides isolated from tarantulas. Oa1a showed the highest identity and similarity with the venom peptide Df1a isolated from the tarantula *Davus fasciatus* in a previous study by us [21], followed by the infamous venom peptide β/ω-Tp1a isolated from the tarantula *Thrixopelma pruriens*, also known as ProTx-I [24], along with other tarantula venom peptides displaying lower identities and/or similarities. Residues differing between Oa1a and Df1a are highlighted in blue boxes in Oa1a. Residues hypothesized to participate in T-type modulation in Df1a are highlighted in green boxes in the primary sequence of Df1a. (**B**,**C**) Molecular modelling of the three-dimensional (3D) structure of Oa1a and Df1a. The C- and N-terminal ends are highlighted in pink, while residues unique to Oa1a (**B**) or Df1a (**C**) are represented by red lines in their respective 3D structures. The cysteine disulphide bonds are represented by yellow sticks. Remarkable variance is observed in loop 2 between these spider peptides.

The primary sequence of Oa1a was compared to the primary sequences of other tarantula venom peptides displaying the highest identity and similarity in the UniProt Database (https://www.uniprot.org) (Figure 6A). Oa1a had the highest identity and similarity with the tarantula peptide Df1a isolated from the venom of the Central American tarantula *Davus fasciatus* in a previous study by us [21], followed by the infamous venom peptide Tp1a isolated from the South American tarantula *Thrixopelma pruriens*, also known as ProTx-I [24,25]. As for these peptides, Oa1a can slow the fast inactivation of Na_V_ channels, but contrariwise Oa1a has no activity on Ca_V_3 channels. This makes Oa1a a unique peptide with useful structural and functional properties allowing us to unravel the pharmacological mechanisms of selectivity of spider peptides targeting the Na_V_ and Ca_V_3 channels. This also suggests that Asian tarantula venom peptides with dual pharmacology for Na_V_ channels are selective for Na_V_ channels and do not act on Ca_V_3 channels, differing from their counterpart tarantulas in Latin America.

The three-dimensional structure of Oa1a was predicted using the AlphaFold 2 algorithm (Figure 6B), along the three-dimensional structure of the venom peptide Df1a (Figure 6C) to allow comparison between their structure–function relationships. These peptides share 85% similarity, with residues differing in Oa1a highlighted in the primary sequence in blue boxes (Figure 6A) and described in Oa1a’s 3D structure (Figure 6B). Oa1a differs significantly in the residues present in loop 2, with Oa1a containing two highly opposite charges in E13 and K14, while Df1a contains the neutral residues Q13 and T14 in these same positions. In addition, loop 4 in Oa1a comprises N23 while Df1a comprises the positive charged residue R23 in the same position. All remaining differences between Oa1a and Df1a are residues with high similarity and likely do not contribute significantly to their distinct pharmacology (Y4 in Oa1a and W4 in Df1a). Based on these observations, we hypothesize Oa1a’s lack of activity at T-type channels is due to the presence of highly charged residues in loop 2 and reduced positive charges in loop 4. The corresponding residues in Df1a likely allowing for activity at T-type channels are highlighted in green boxes in the primary sequence of Df1a (Figure 6A).

## 3. Discussion

Venom peptides have emerged as structurally diverse and mechanistically precise modulators of ion channels, reflecting millions of years of evolutionary selection for prey capture and predator deterrence [26,27]. Among these, spider venoms are enriched in inhibitory cystine-knot (ICK) scaffolds that confer both structural rigidity and functional adaptability, enabling high-affinity interactions with voltage-gated sodium (Na_V_), potassium (K_V_), and calcium (Ca_V_) channels [28]. In contrast, snake venoms are particularly notable for their three-finger toxins (3FTx), which lack the ICK motif but display extensive conformational plasticity, allowing them to target ligand-gated channels, such as nicotinic acetylcholine receptors as well as selected voltage-gated channel subtypes [5].

Within spider-derived toxins, the Oa peptide illustrates the capacity of subtle sequence and structural modifications to shape channel specificity and gating modulation. Comparative analyses of the activity of the synthetic and recombinant forms of Oa1a with Df1a and related members of the spider peptides family demonstrate that Oa peptides occupy overlapping, yet distinct pharmacological space at Na_V_ channels, with differential sensitivity across mammalian subtypes linked to variations in electrostatic surface potentials and hydrophobic residues at the channel-binding interface [21]. This indicates that Na_V_ subtype selectivity in related spider peptides is not scaffold-determined but is instead tuned by discrete residue substitutions that influence interactions with voltage-sensors in domains II and IV.

These contrasts are further accentuated when considering the pharmacology of spider peptides such as ProTx-III and Tap1a [16,19,22]. These peptides display divergent binding determinants relative to Oa1a and Df1a, with evidence for distinct mechanisms of gating modification and toxin–channel interaction [7,19]. Structural studies indicate that the spatial arrangement of disulfide bonds and surface loops confers differential access to Na_V_ channel binding sites, accounting for marked differences in subtype selectivity and state dependence [7]. Collectively, these observations suggest that Na_V_-toxin interactions are governed not only by the global cystine-knot framework but also by scaffold-specific conformational constraints that regulate the engagement with the channel. While studies employing native spider peptides are highly valuable and represent the ideal approach for early-stage discovery research, their application is often limited by the scarce quantities of crude venom available. Consequently, most investigations have relied on synthetic or recombinant versions of these peptides to enable more extensive pharmacological characterization and structural analyses. [16,21,22].

The unique pharmacological properties of these venom-derived peptides are of direct translational interest. For example, spider and scorpion Na_V_ channel blockers with selectivity for nociceptor-enriched subtypes, such as Na_V_1.7 and Na_V_1.8, have demonstrated analgesic efficacy in preclinical models of pain, including neuropathic pain, while minimizing off-target effects on cardiac and skeletal muscle isoforms [16,22,29]. Similarly, K_V_ and Ca_V_-targeting toxins show promise in modulating excitability in epilepsy, ischemia, and neurodegeneration [30,31,32]. Several venom peptides have advanced to preclinical or clinical development, either as unmodified peptides or as templates for rationally engineered analogues optimized for potency, stability, and safety [5,19]. Thus, venom peptides constitute not only molecular probes for dissecting ion channel physiology but also structurally validated starting points for therapeutic innovation [5,33].

Systematic structure–function studies that employ residue substitution, domain grafting, and computational modelling are needed to delineate the contributions of individual residues and loop conformations to channel binding and gating modulation [19,34]. Such efforts must be coupled with pharmacological evaluation at physiologically relevant ion channel isoforms, particularly those implicated in human disease pathophysiology, to maximize translational relevance. Moreover, elucidating the secondary and tertiary structures of spider peptides produced synthetically or through recombinant expression would provide valuable insights into their structure–function relationships.

Our group’s recent work highlights the utility of integrating high-throughput screening (HTS) platforms with advanced electrophysiological, structural, and computational approaches; we have identified novel peptide modulators with refined selectivity and drug-like properties [16,19]. These developments underscore the need of combining broad-scale venomic exploration with mechanistically driven pharmacology, thereby accelerating the translation of venom peptides into precision therapeutics targeting ion channel dysfunction.

In conclusion, the comparative analysis of venom peptide families highlights how evolutionary variation in structural motifs translates into pharmacological mechanism diversity in modulating ion channels. Continued integration of structural biology, pharmacology, and venomics-driven discovery will be essential to unlock the pharmacological and therapeutic potential of these molecules. Such multidisciplinary approaches promise to transform venom peptides from natural toxins into next-generation tools and drug leads for ion channel–related disorders.

## 4. Conclusions

This study underscores how evolutionary diversification of venom peptide structures drives their distinct pharmacological mechanisms at ion channels. The Oa1a peptide exemplifies how subtle sequence and structural variations fine-tune Na_V_ subtype selectivity, revealing that specific residue substitutions, rather than the global scaffold, dictate channel interactions. Integrating structural biology, pharmacology, and venomics-guided discovery will be crucial to fully harness these peptides as molecular tools and therapeutic leads, transforming natural toxins into precision modulators for ion channel–related diseases.

## 5. Materials and Methods

### 5.1. Reagents

Crude venom from *Ornithoctonus aureotibialis* was collected by electrostimulation of the chelicerae [35], lyophilized, and stored at −80 °C. For further analysis, venom was weighed and re-constituted in an appropriate buffer.

### 5.2. Cell Culture

Unless otherwise specified, all cell culture reagents were obtained from Life Technologies (Carlsbad, CA, USA). The human neuroblastoma cell line SH-SY5Y (ATCC CRL-2266; Manassas, VA, USA) was maintained in RPMI medium supplemented with 15% fetal bovine serum (FBS) and 2 mM L-glutamine. HEK293 cells stably expressing human Na_V_ channel isoforms together with the β1 auxiliary subunit (SB Drug Discovery, Glasgow, UK) were propagated in Minimal Essential Medium (MEM; Sigma-Aldrich, St. Louis, MO, USA) containing 10% FBS, 100 U/mL penicillin, 100 µg/mL streptomycin, 2 mM L-glutamine, and selective antibiotics (blasticidin, geneticin, and zeocin) at concentrations recommended by the supplier. HEK293T cells expressing the human Ca_V_3.2 channel (a generous gift from Prof. Emmanuel Bourinet, Institut de Génomique Fonctionnelle, Université de Montpellier, France, and Prof. Edward Perez-Reyes, University of Virginia School of Medicine, USA) were grown in Dulbecco’s MEM supplemented with 10% FBS, 100 U/mL penicillin, 100 µg/mL streptomycin, and 750 µg/mL geneticin.

All cell lines were cultured at 37 °C in a humidified incubator with 5% CO_2_. Subculturing was performed every 3–4 days at a 1:5 split ratio using 0.25% trypsin-EDTA for SH-SY5Y and Na_V_-expressing HEK293 cells, and Versene solution for Ca_V_3.2-expressing cells.

### 5.3. Venom Fractionation

Crude venom from *Ornithoctonus aureotibialis* (1 mg) was reconstituted in 100 µL of Milli-Q water containing 0.05% trifluoroacetic acid (TFA; Auspep, VIC, Australia) and 5% acetonitrile (ACN; Sigma-Aldrich, St. Louis, MO, USA). The solution was clarified by centrifugation at 20,000× *g* for 10 min to eliminate insoluble material. Venom components were then separated by reverse-phase high-performance liquid chromatography (RP-HPLC) on a C18 column (Vydac, 4.6 × 250 mm, 5 µm; Grace Discovery Sciences, Columbia, MD, USA). Elution was performed with a linear gradient of Solvent B (90% ACN in 0.045% TFA) against Solvent A (0.05% TFA in Milli-Q water). The separation began with an isocratic phase of 5% Solvent B for 5 min, followed by a gradient from 5% to 20% B between 5 and 10 min, and a further increase from 20% to 40% B over the next 40 min. The column flow rate was maintained at 0.7 mL/min, and eluate fractions (0.7 mL each) were collected, freeze-dried, and stored at −20 °C until further analysis.

### 5.4. Calcium Influx Assay

Venom fractions were screened against hNa_V_1.7 in SH-SY5Y cells using a Fluorescent Imaging Plate Reader (FLIPR Penta; Molecular Devices, San Jose, CA, USA) as previously described [22]. Briefly, SH-SY5Y cells were plated at 40,000 cells per well in 384-well flat clear-bottom black plates (Corning, Corning, NY, USA) and cultured at 37 °C in a humidified 5% CO_2_ incubator for 48 h before commencing assays. Cells were loaded with 20 μL per well of Calcium 4 dye (Molecular Devices) reconstituted in assay buffer containing (in mM) 140 NaCl, 11.5 glucose, 5.9 KCl, 1.4 MgCl_2_, 1.2 NaH_2_PO_4_, 5 NaHCO_3_, 1.8 CaCl_2_, and 10 HEPES pH 7.4 and incubated for 30 min at 37 °C in a humidified 5% CO_2_ incubator. Fluorescence responses were recorded at excitation wavelength of 470–495 nm and emission 515–575 nm for 10 s to set the baseline, 600 s after addition of 1 to 0.001 μg.ml^−1^ venom, and for further 300 s after addition of 3 μM veratridine and 30 nM of the scorpion toxin OD1. Tetrodotoxin (TTX) at 1 μM was used as a Na_V_ inhibition control.

### 5.5. Mass Spectrometry and Amino Acid Sequencing

Peptide masses were identified through ESI-MS (Shimadzu, Kyoto, Japan) or MALDI-TOF MS using a Model 5800 Proteomics Bioanalyzer (Applied Biosciences, Waltham, MA, USA). For MALDI-TOF MS, RP-HPLC fractions, and α-cyano-4-hydroxycinnamic acid (CHCA) (Sigma-Aldrich), matrices (7.5 mg/mL 23 in 50/50 acetonitrile/H_2_O, 0.1% TFA) were mixed (1:1 *v*/*v*) and spotted onto a MALDI plate. Positive reflector mode was used to obtain mass spectra. Monoisotopic [M + H] [7] + ion masses are reported throughout the thesis. Data Explorer software (4.9, Applied Biosystems, Foster City, CA, USA) was used for analyzing mass spectral data. After confirmation of peptide mass using ESI-MS/MALDI-TOF MS, fractions containing the peptides were lyophilised and stored at −20 °C until further use.

N-terminal sequencing was outsourced to the Australian Proteome Analysis Facility. Briefly, peptides were reduced using dithiothreitol (25 mM) and incubated at 56 °C for 30 min. The samples were then alkylated using iodoacetamide (55 mM) at room temperature for 30 min. The samples were purified via RP-HPLC using a Zorbax 300SB-C18 column (3 × 150 mm) (Agilent, Santa Clara, CA, USA). The target peaks of interest were identified and reduced to minimal volume under vacuum. Approximately 90% of the collected sample was loaded onto a pre-cycled Biobrene-treated disc. The samples were subjected to 38–42 cycles of Edman degradation using an ABI 494 Procise Protein Sequencing System (Applied Biosystems, Waltham, MA, USA).

### 5.6. Solid-Phase Peptide Synthesis and Oxidative Folding

Linear Oa1a-NH_2_ and Oa1a-OH were assembled via Fmoc-SPPS at 0.1 mmol scale with a Liberty Prime automatic synthesizer (CEM, Charlotte, NC, USA). For both peptides either Fmoc-Phe-Wang or Protide Rink-amide resin in the case of C-terminal amidation were used, with the following side-chain protecting groups used in the synthesis: Arg (2,2,4,6,7-pentamethyldihydrobenzofuran-5-sulfonyl), Asp (O-3-methyl-pent-3-yl), Glu (*tert*-butyl ester), Cys, Gln (trityl), Lys/Trp/His (*tert*-butyloxycarbonyl), and Ser/Thr/Tyr (*tert*-butyl). Removal of the Fmoc protecting group was achieved via 25% pyrrolidine/dimethylformamide (DMF), while DMF, diisopropylcarbodiimide and Oxyma Pure (1:2:1 equivalents [eq]) were used for coupling Fmoc-amino acids (5 eq) at 105 °C. After main-chain assembly and final Fmoc deprotection, the resin was thoroughly washed with dichloromethane/MeoH prior to cleavage with 92.5% trifluoroacetic acid (TFA), 2.5% triisopropylsilane, 2.5% 3,6-dioxa-1, 8-octaneidithiol, and 2.5% H_2_O at 40 °C for 40 min. Cleaved peptide products were precipitated with ice-cold diethyl ether and subsequently dissolved in ACN/H_2_O (0.3:1) and lyophilized. Crude peptides were purified via reversed-phase high-performance liquid chromatography (RP-HPLC) with a Zorbax 300SB–C18 preparative column (21.2 × 150 mm; 7 μm particle size; 300 Å pore size; Agilent Technologies) with a gradient of 10–60% solvent B (90% ACN, 10% H_2_O, 0.043% TFA) over 50 min. Peptide peaks were identified using ESI-MS in positive ion mode using an API2000 triple-quadrupole mass spectrometer (AB SCIEX Australia, Mulgrave, VIC, Australia). Peaks with masses matching those of reduced Oa1a-NH_2_ and Oa1a-OH were lyophilized prior to oxidation.

Purified reduced peptides were dissolved in the oxidative folding buffer (Tris 0.1 M, 1.35 M urea, 1.5 mM reduced glutathione, 0.15 mM oxidized glutathione; pH 8) at a concentration of 0.1 mg/mL and stirred at 4 °C for 48 h, then the reaction was quenched by the addition of 0.1% TFA. Oa1a-NH_2_ and Oa1a-OH were then further purified via Prep-HPLC as previously described [21]. Cysteine oxidation was confirmed via a 6 Da reduction in mass and a shift in HPLC retention time, while isolation of the correct isomer was determined via biological activity on Na_V_1.7. Peptide purity was determined via peak integration on an analytical UPLC (Phenomenex Aeris XB-C18 column; 250 × 4.6 mm; particle size 3 μm; 100 Å pore size), while yields were determined via absorbance at 280 nm measured via a Nanodrop spectrometer based upon the predicted extinction coefficient.

### 5.7. Recombinant Expression

A synthetic gene encoding the Oa1a peptide, optimized for *Escherichia coli* expression, was synthesized and inserted into the pLICC plasmid (GeneArt, Life Technologies, Carlsbad, CA, USA). The construct included a MalE signal peptide for periplasmic targeting, an N-terminal His_6_–maltose-binding protein (MBP) fusion tag, and a tobacco etch virus (TEV) protease cleavage site positioned upstream of the Oa1a coding sequence. Peptide expression followed previously established procedures [22]. In brief, *E. coli* BL21(DE3) cells transformed with pLICC-Oa1a were cultured in Luria–Bertani (LB) broth containing 100 µg/mL ampicillin at 37 °C with shaking at 280 rpm until the optical density at 600 nm (OD_600_) reached approximately 0.8. The incubation temperature was then reduced to 16 °C, and protein production was induced with 0.5 mM isopropyl β-D-1-thiogalactopyranoside (IPTG; Astral Scientific, Taren Point, NSW, Australia). After 16 h of induction, cells were collected by centrifugation, and the resulting pellets were resuspended in TN buffer (50 mM Tris, 300 mM NaCl, 15 mM imidazole, pH 8.0) before lysis using a TS Series Benchtop Cell Disruptor (Constant Systems Ltd., Daventry, UK).

Soluble lysate was collected by centrifuging and the fusion protein (His6-MBP-peptide) purified via nickel ion affinity chromatography using charged Ni-NTA superflow resin (QIAGEN), and the fusion protein was eluted with TN buffer containing 300 mM imidazole. Buffer exchange of eluted fusion peptide was carried out using TN buffer and a centrifugal filtration device (Amicon, Ultra-30K, Millipore, MA, USA). Redox buffer containing reduced glutathione (6 mM GSH) and oxidized glutathione (0.6 mM GSSG) was used for TEV cleavage for 120 h at 4 °C.

Post-cleavage, the His6-MBP tag and TEV protease were precipitated by acidifying the samples with 1% trifluoroacetic acid (TFA) (Auspep, Tullamarine, VIC, Australia) followed by the addition of HPLC solvent B (0.05% TFA, 90% acetonitrile (Sigma-Aldrich) to the sample to a final concentration of 5%. After incubation for 30 min at room temperature (RT), the precipitated products were removed by centrifugation at maximum speed for 5 min before purification of the peptide using reversed phase high-performance liquid chromatography (RP-HPLC).

RP-HPLC purification of rOa1a was carried out using an analytical C18 column (Phenomenex, Kinetex, 250 Å, 4.6 mm, 5 μm particle size) and a flow rate of a 1 mL/min in solvent B containing 0.43% trifluoracetic acid (TFA) in 90% acetonitrile and solvent A containing 0.5% TFA in water. A linear gradient of 10–80% solvent B over 65 min was used for purification. Peak fractions were collected manually, and peptide mass was identified using matrix-assisted laser desorption/ionization-time of flight (MALDI-TOF) mass spectrometry. Fractions with masses corresponding to the predicted mass of the recombinant peptide were pooled, lyophilised, and resuspended in water.

### 5.8. Whole-Cell Patch Clamp Electrophysiology

Sodium channel currents were recorded from HEK293 cells expressing human Na_V_ isoforms together with the β1 auxiliary subunit (SB Drug Discovery, Glasgow, UK) using the QPatch 16X automated whole-cell patch-clamp platform (Sophion Bioscience A/S, Ballerup, Denmark). The external recording solution contained (in mM) the following: 140 NaCl, 3 KCl, 1 CaCl_2_, 1 MgCl_2_, 0.1 CdCl_2_, 20 tetraethylammonium chloride (TEA-Cl), and 5 HEPES; pH was adjusted to 7.3 and osmolarity to 320 mOsm. The internal (pipette) solution consisted of (in mM) the following: 140 CsF, 10 NaCl, 10 HEPES, and 1/5 EGTA/CsOH, also adjusted to pH 7.3 and 320 mOsm. Cells were voltage-clamped at a holding potential of −80 mV, and inward Na^+^ currents were evoked by 20 ms depolarizing pulses to 0 mV following a 200 ms hyperpolarizing prepulse to −120 mV. To obtain concentration–response curves, cells at holding potential were incubated for 5 min with increasing concentrations of Oa1a peptides. For determining the potency of Oa1a peptides to inhibit peak current, the maximum peak current within the 20 ms at 0 mV was measured. For determining the potency of Oa1a-NH_2_ to slow fast inactivation, peak current values were measured at 20 ms of the 0 mV depolarization step. For determining the potency of Oa1a-NH_2_ to slow fast inactivation, peak current values were measured at 20 ms of the 0 mV depolarization step. Ion currents (*I*_Na_) were plotted as *I*/*I*_max_, where *I* = current value and *I*_max_ = highest current value within the experiment.

The voltage dependence of steady-state activation was assessed by recording sodium currents evoked by depolarizing voltage steps ranging from −110 mV to +80 mV in 5 mV increments. Peak conductance (*G*_Na_) was derived using the equation *G* = *I*/(*V* − *V*_rev_), where *I* denotes the measured current, *V* the applied membrane potential, and *V*_rev_ the sodium reversal potential. To evaluate the voltage dependence of steady-state fast inactivation, a two-pulse protocol was used. Cells were conditioned with 500 ms pre-pulses at potentials between −130 mV and −10 mV (10 mV increments), followed by a 20 ms test depolarization to 0 mV to elicit sodium currents. The steady-state fast inactivation currents were plotted as *I*/*I*_max_, where *I* = current value and *I*_max_ = highest current value within the experiment. The voltage-dependence of steady-state activation and inactivation were determined either in the absence of peptide or following 5 min exposure to peptide at the respective IC_50_ concentration at the Na_V_ subtype analyzed to streamline the investigation using an effective single concentration as previously described by us [16,19,21,22].

For off-rate measurements, cells at a holding potential −80 mV were incubated with peptide for 10 min at a concentration equivalent to 10 times their IC_50_ for the Na_V_ subtype being analyzed, followed by saline washes. Na^+^ currents were assessed every 10 s utilizing a holding potential −80 mV and currents elicited by 20 ms voltage step to 0 mV from a −120 mV conditioning pulse applied for 200 ms. Ion currents (*I*_Na_) plotted as *I*/*I*_max_, where *I* = current value and *I*_max_ = highest current value within the experiment.

Ca_V_3.2 channel currents were recorded from HEK293 cells expressing hCa_V_3.2 using an automated QPatch 16X platform (Sophion Bioscience A/S). The extracellular solution comprised (in mM) 5 CaCl_2_, 0.5 MgCl_2_, 10 HEPES, 157 TEA-Cl at pH 7.3, and 320 mOsm, and the intracellular solution comprised (in mM) 140 CsF, 1 EGTA, 10 HEPES, 10 NaCl at pH 7.3, and 320 mOsm. Cells were maintained at a holding potential of −90 mV and Ca^2+^ currents elicited by 60 ms voltage steps to −30 mV from a −120 mV conditioning pulse applied for 60 ms. To obtain concentration–response curves, cells at holding potential were incubated for 5 min with increasing concentrations of Oa1a peptides. Ion currents (*I*_ca_) were plotted as *I*/*I*_max_, where *I* = current value and *I*_max_ = highest current value within the experiment.

### 5.9. Molecular Modelling

Venom peptides in this study had their three-dimensional (3D) structures predicted using the AlphaFold 2 algorithm [36]. All 3D structures displayed were from unrelaxed models ranked 1 for each peptide prediction. The 3D structures were visualized and analyzed using PyMol [37].

### 5.10. Data Analysis

For calcium influx experiments, bioactivity comparisons between venom fractions and controls were based on the difference between the maximal and minimal fluorescence intensity values obtained from single-replicate assays. Electrophysiological data analysis was conducted using GraphPad Prism (version 8; GraphPad Software, San Diego, CA, USA). Nonlinear regression was applied to fit the concentration–response relationships using a variable Hill slope: log(inhibitor) versus normalized response for determining IC_50_ values, and log(agonist) versus normalized response for EC_50_ calculations. Reversibility and dissociation kinetics were analyzed by fitting data with a single-phase exponential decay model, while voltage-dependence of steady-state activation and inactivation parameters were derived from Boltzmann sigmoid fits of normalized conductance (*G*/*G*_max_) or current (*I*/*I*_max_) values to determine the half-activation or half-inactivation voltages (V_50_). Data are expressed as mean ± SEM, where *n* represents the number of individual cells analyzed.

## Figures and Tables

**Table 1:** Na_V_ channel subtype selectivity of peptides rOa1a, sOa1a-NH_2_, and sOa1a-OH evaluated via automated patch-clamp electrophysiology of cell lines expressing Na_V_s. Na^+^ currents were measured before and after the addition of peptides, and the calculated IC_50_ and ratio OH/NH_2_ are indicated for each Na_V_ subtype. Data are from 3 to 9 independent experiments.

hNa_V_ Subtype	rOa1a IC_50_ (nM) Mean ± SEM	sOa1a-NH_2_ IC_50_ (nM) Mean ± SEM	sOa1a-OH IC_50_ (nM) Mean ± SEM	Ratio OH/NH_2_
Na_V_1.1	1494 ± 182	17 ± 1	274 ± 18	16
Na_V_1.2	640 ± 85	154 ± 7	>3000	na
Na_V_1.3	899 ± 20	162 ± 8	>3000	na
Na_V_1.4	1031 ± 48	2289 ± 77	>3000	na
Na_V_1.5	1002 ± 31	192 ± 4	1266 ± 22	6.6
Na_V_1.6	792 ± 19	223 ± 18	568 ± 38	2.6
Na_V_1.7	432 ± 25	25 ± 0.4	410 ± 23	16

**Table 2:** Mode of action of Oa peptides on select Na_V_ channel subtypes. Off-rate evaluations investigated the reversibility properties of synthetic Oa1a-OH and Oa1a-NH_2_ on Na_V_1.1, with respective calculated dissociation time (off-rate) expressed in minutes. The voltage-dependence of steady-state inactivation and activation were measured for Na_V_1.3, with the V_50_ values calculated in the absence and presence of sOa1a-NH_2_ and ΔV_50_ of steady-state inactivation calculated. The voltage-dependence of steady-state inactivation and activation were measured for Nav1.7, with the V_50_ values calculated in the absence and presence of sOa1a-NH_2_. The potency of sOa1a-NH_2_ and rOa1a in slowing the fast inactivation of Na_V_1.3 were calculated, with EC_50_ values calculated in nM. Data are from 3 to 5 independent repeats. ND = not determined.

hNa_V_ Subtype	sOa1a-NH_2_	sOa1a-OH	rOa1a
Na_V_1.1	Off-rate 4.6 min (95% CI = 4.27 to 4.97 min)	Off-rate 3.56 min (95% CI = 3.35 to 3.80 min)	ND
Na_V_1.3	Steady-state inactivation (mean ± SEM) Control V_50_ = −71 ± 0.71 mV Peptide V_50_ = −57 ± 1.4 mV ΔV_50_ = 14 mV Steady-state activation (mean ± SEM) Control V_50_ = −21 ± 0.4 mV Peptide V_50_ = −26 ± 1 mV Slowing fast inactivation (mean ± SEM) EC_50_ = 22.9 ± 1.8 nM	ND	Slowing fast inactivation (mean ± SEM) EC_50_ = 109.6 ± 7.2 nM
Na_V_1.7	Steady-state inactivation (mean ± SEM) Control V_50_ = −66 ± 0.7 mV Peptide V_50_ = −69 ± 1.1 mV Steady-state activation (mean ± SEM) Control V_50_ = −28 ± 0.5 mV Peptide V_50_ = −30 ± 0.3 mV	Steady-state inactivation (mean ± SEM) Control V_50_ = −66 ± 0.3 mV Peptide V_50_ = −71 ± 0.8 mV Steady-state activation (mean ± SEM) Control V_50_ = −25 ± 0.7 mV Peptide V_50_ = −22 ± 1.7 mV	ND

## Data Availability

The original contributions presented in this study are included in the article/Supplementary Materials. Further inquiries can be directed to the corresponding author.

## Supplementary Materials

The following supporting information can be downloaded at: https://www.mdpi.com/article/10.3390/toxins17110561/s1, Figure S1: Effect of the synthetic Oa1a peptides on Ca_V_3.2 channel.

## Abbreviations

The following abbreviations are used in this manuscript:

ICK	Inhibitory Cysteine Knot
Na_V_	Voltage-gated sodium channel
Ca_V_	Voltage-gated calcium channel
K_V_	Voltage-gated potassium channel
HEK293	Human Embryonic Kidney 293 cell line
TEV	Tobacco Etch Virus
RP-HPLC	Reverse Phase High Performance Liquid Chromatography
