# Supplementary material for: Venom Peptides Across Asian and American Tarantulas Utilize Dual Pharmacology to Target Activation and Fast Inactivation of Voltage-Gated Sodium Channels
Source: Toxins (Basel). 2025 Nov 14;17(11):561. doi: 10.3390/toxins17110561 (PMC12656268; doi:10.3390/toxins17110561)
Supplement: Supplementary file 1 [file toxins-17-00561-s001.zip › toxins-3948756-supplementary.pdf]

Supplementary Materials: Venom peptides across Asian and American tarantulas utilize dual pharmacology to target activation and fast inactivation of voltage-gated sodium channels

Amatulla S. Nashikwala, Charan Kotapati, David A. Eagles, Richard J. Lewis and Fernanda C. Cardoso

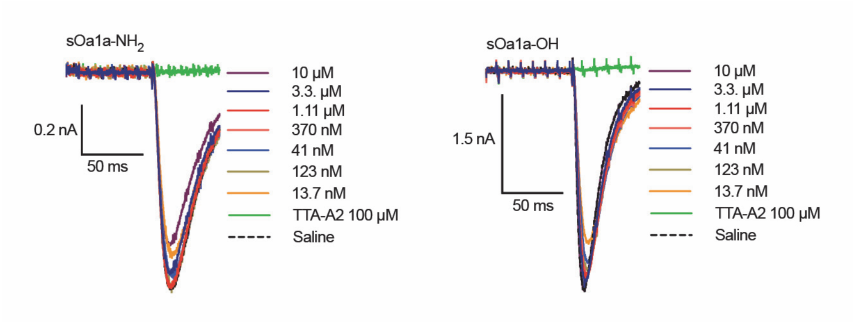

Figure S1. Effect of the synthetic Oa1a peptides on CaV3.2 channel. We tested both synthetic forms of Oa1a-NH2 and Oa1a-OH on the T-type channel subtype CaV3.2 using automated whole-cell patch clamp electrophysiology and a close-state voltage protocol. We did not observe activity on the CaV3.2 channel at up to 10 µM tested.
